# Identity and psychological ownership in chronic illness and disease state

**DOI:** 10.1111/j.1365-2354.2010.01220.x

**Published:** 2011-03

**Authors:** W Karnilowicz

**Affiliations:** School of Social Sciences and Psychology, Victoria UniversityMelbourne, Victoria, Australia

**Keywords:** cultural, prostate cancer, psychological, international

## Abstract

Psychological ownership is rarely considered in health discourse related to chronic illness or disease state. Construction of identity is an important consideration within this framework. This autoethnographic study explores psychological ownership and identity related to prostate cancer and chronic illness. Conclusions about the nature of psychological ownership and identity were gathered from the relevant literature and personal experience. Themes include the patient–healthcare professional relationship and that psychological ownership is personal and grounded in an individual's sense of identity, control and perceived capacity to control illness or disease. Personal reflection through autoethnography guides discussion of psychological ownership and identity.

## INTRODUCTION

At 50 years of age and largely as a result of media advertising and warnings in Australia associated with various forms of cancer, in May of 2004 I went into my local general practitioner's (GP) surgery. None-the-wiser and among other standard tests the GP completed a rectal and blood (PSA – prostate-specific antigen) examination for prostate cancer. I feared little as there were an absence of real physical signs of medical problems prior to the tests and the result of the rectal examination was clear. However, my daily thoughts and activities were disrupted some days after on receiving a phone call in my university office requesting that I come back into the GP's surgery to discuss my PSA result. There were alarm bells. I rushed from work immediately and my mind reflected throughout that 1-h journey. Within our meeting the doctor expressed concerns with my relatively high PSA score and suggested I should have further examinations to confirm the existence of prostate cancer. Further tests over a period of months, including a biopsy and a series of scans through the Department of Urology at the Royal Melbourne Hospital, subsequently confirmed that I had prostate cancer.

Fitness and health are integral components of my identity and the news that I had cancer was initially devastating. It affected me in more ways and more deeply than I thought possible. The disease invaded every part of my psychological self and was borne as much out of a fear of the unknown as the known. I was overwhelmed by the disease and life did not extend beyond the near and immediate days. The dates of the many medical appointments and additional tests were a ‘blur’ and every little back pain or slight illness was a sign for the worst. In time, the disease came to own me. I reacted in seeking to know everything about it and to understand its nature and character. I read extensively through the relevant and not so relevant available information via the Internet and university journal resources, communicated with fellow victims and most importantly talked extensively with the urologists. It was an endeavour to take control away from the disease and to ‘own’ it and this activity invaded my every thought and action. There were many unanswered questions and even more concerns. Among other factors, including family and friends, my relationship with the various GPs and specialist urologists was central to the understanding and handling of the disease. Following a number of discussions with the urologists, the considered best course of action was a radical prostatectomy subsequently undertaken in January of 2005. My mind particularly from the time that I was first informed of the real possibility of having prostate cancer to the time that it was confirmed and then the time up to, involving and surrounding the radical prostatectomy was fully devoted to dealing with the cancer and its associated physical and psychological effects. Fortunately, a pathology test of the prostate gland following the radical prostatectomy indicated that the cancer was confined to the prostate. I was relieved although subsequent PSA tests monitoring my progress over the next 12 months and the associated test result was met with trepidation. Nonetheless, this was a journey of discovery. In particular, there was the epiphanic moment of diagnosis as a significant life event that led down the path of determining psychological ownership as an important part of recovery and identity reconstruction. The journey also involved an assessment and interpretation of the important characteristics and nature of the reality of the patient–practitioner relationship.

Psychological ownership has not been studied extensively in contemporary health and cultural psychology. Instead it has been investigated mainly in relation to consumerism and principally in reference to ownership of possessions (Pierce *et al*. 2003). However, the notions of ownership are relevant to the extent that disease state or chronic illness may also be considered a reference ‘target’ (Pierce *et al*. 2003). For example, Pierce *et al*. (2003) argued ownership manifests itself in the emotion and meaning frequently associated with ‘my’ or ‘mine’ and further suggested that ownership is a reflection of the relationship between the individual and the target object. This is a complex state comprising self-awareness, thoughts and beliefs.

Pierce *et al*. (2003) maintained that individuals are motivated to define and express their self-identity. Self-identity is also the boundary between the individual and society as the individual develops a sense of self-identity through viewing themselves through the eyes of society (Clarke & James 2003; Pierce *et al*. 2003). Therefore, ownership becomes a symbol of the self as it satisfies the basic needs of self-identity (Pierce *et al*. 2003). The idea of ownership as a symbol of the self is a fundamental sociological concept (Clarke & James 2003). It is a dynamic and fluid process of identification and its meaning is grounded in an individual's self-identity. Within this framework, identification with a chronic illness or disease state is dynamic and emergent. It changes as the individual reflexively interprets the identification that ‘self’ and others bestow upon him or her (Clarke & James 2003).

The experience of identifying with and owning an illness or disease is fixed in the idea of control. Ownership therefore considers the fusing of the target with the self (Pierce *et al*. 2003). The greater the level of control, the more likely control is experienced psychologically as part of self. Along with control, responsibility is also grounded in identification and ownership. Improving, maintaining or protecting one's identity may result in an enhanced sense of responsibility (Pierce *et al*. 2003) towards the illness or disease state.

While Pierce *et al*. (2003) argued for two schools of thought in relation to the origin of the experience of ownership including the biological perspective and a social constructionist perspective, psychological ownership in this instance is considered a social construct. The social constructionist perspective of ownership focuses on the influences of cultural practices and symbols enacted within social interactions and in social settings. Included within this construction is the reality constructed through the relationships between the practitioner and the person with the disease state. The approach contends that social interactions within a social setting form cultural systems (Bock 1988). It is psychosocial-cultural as culture is central to not only the experience of ownership but also in that lived cultural reality within the practitioner–client interaction. The term psychosocial recognises that there is always a close and ongoing interaction between an individual's psychological state and his or her social environment (Shaw 1999).

## METHOD

Psychological ownership and identity was investigated through autoethnography. Autoethnography is a form of narrative and research connecting the personal to the cultural and places the self within a social context (Reed-Danahay 1997). It is a method not limited to a subjective account of experience but also portrays multiple aspects of a social phenomenon (Spry 2001). It is a narrative of personal experience and is also used in this instance to confront dominant forms of representation and power in an attempt to make room for marginalised spaces (Tierny 1998). For example, Foucault (1980) proposed that professionals and specialists in possession of expert knowledge have the power to control over those with less power and are able to regulate society by prescribing actions and discipline. This power differential is especially evident in the health industry (Tang & Anderson 1999) and enacted within the associated hospital consulting rooms, local doctors surgeries and health clinics. Within this model it is important to consider that professionals and patients often interpret the reality of illness or disease differently. Therefore, the analysis of illness or disease from the perspective of the less powerful through an examination of a patients' lived experience of their illness (Sweeney *et al*. 2001) is as necessary as it is valuable. Autoethnography enables one to examine meaning through their lived experience and this is an investigation of the personal experience of psychological ownership and how it is constructed when confronted by a life-threatening disease. Included is an account of the nature of the practitioner–patient relationship, which is importantly considered within ownership.

While autoethnography promotes this search for meaning, it does not prescribe method. Instead, autoethnography falls on a continuum between art and science (Ellis 1999). The approach is subjective and personal although one's self-revelations necessarily involve revelations of others (Ellis 2007). Consequently, there is the need for considered self-censorship (Philaretou & Allen 2006) within an ethic of care (Ellis 2007).The task is to know and understand what psychological ownership and identity means. In short, it is an examination of the construction of identity and ownership. This study examines a socially constructed reality interpreted from the perspective of one who has experienced a life-threatening disease. It is not meant to be self-indulgent or selfish but serves to present a unique albeit shared view of that ‘reality’. In short, self as an autoethnography is the autobiographical narrative individuals use to explain and account for their personal life world (Clarke & James 2003) within a cultural framework. The validity of this narrative is determined by the degree to which the reader as participant accepts it as truthful and meaningful.

## DISCUSSION

Notions of identity and psychological ownership are inextricably linked. Identity is socially prescribed and constructed within a system of symbols, values and beliefs represented and enacted through an individual's behaviour and associated cognitive processes. These symbols, values and beliefs in turn are mediated through the active experience of psychological ownership (Pierce *et al*. 2003). This process constitutes a ‘single’ reality constructed as a singular ‘whole’ with the cultural, the social and the self considered as one.

With a life-threatening disease or chronic illness, the process of psychological ownership is often instigated by an epiphany as a sudden revelation, turning point, significant life event, experience, critical juncture or realisation within a significant life event (King *et al*. 2003). In this instance the epiphanic moment was a response to the initial diagnosis, the latter represented and expressed through the words and actions of the GP in the first instance and then followed by the urologist. As was the case in this instance, epiphany moments are rarely planned, often unpredictable and uncontrollable and may result in a fundamental shift in the meaning, direction or purpose of one's life (Rasmussen *et al*. 2007). In short, the experience alters an individual's concept of self and view of life and leads to new levels of insight (Goud 1995).

Culture and the associated social context are the bases for psychological ownership related to illness and disease state. In this sense, the essence of health care is a cultural construction borne out of a physical and physiological reality developed from beliefs about the nature of disease and the human body. However, different cultures with varying concepts, aetiologies and definitions of health have different systems and methods for defining good health and understanding treatment (Farrell *et al*. 2004). Therefore, the management of illness is multidimensional and involves a complex interplay of personal, cultural and social circumstances (Townsend *et al*. 2006). More to the point is that culture is embedded within an individual's perceptions and processes in interpreting information (Matsumoto & Juang 2004) and in the way meanings are formulated (Ratner 1997). In particular, cultural beliefs influence an individual's sense of control and self-efficacy related to the illness or disease state. Nonetheless, the biomedical paradigm involves experts who take control, problematise, label and treat according to a prescribed symptom(s). The cause of the problem and a patient's identity are rarely considered or addressed. Chronic illnesses are therefore objectified and often hidden behind the doors and within the walls of the clinician's office.

The systemic aspects of healthcare professional and patient relationships are positioned within interpersonal communication and the power of discourse. Different categories of relationship exist (e.g. patient-driven, collaborative or authoritarian) and communication styles vary in level of assertiveness. Education also provides greater authoritative credibility. Discussions with health professionals are largely ‘expert’-based associated with immediate imbalances within an authoritative communication relationship. However, personal experience and knowledge of the disease and disease state become important in destabilising established power differences in medical practice and enhance feelings of ownership. For example, Winkelman *et al*. (2005) noted that patients' realities, their values and priorities, are not reflected in medical records and suggested that if patients keep a record of their illness experiences from their own perspective this will enhance their sense of illness ownership. In addition, illness or disease and notions of ownership also reside within family history and expectations concerning illness. Taboos, myths and rituals can influence communication and act as sources of knowledge and action.

Discourses from critical and feminist postmodern perspectives challenge the traditional biomedical model and question science's claims of universal truths and draw attention to how scientific discourse and practices are and should be embedded in a social context. The postmodernists critique and question the hegemony of biomedicine and expert knowledge (Tang & Anderson 1999). My experience of diagnosis and the first thought of being ‘diseased’ led to a motivated, compulsive and focused investigation of everything that was prostate cancer. This compulsive and determined behaviour led to a largely unintended but more balanced interpersonal relationship with the urologist. While acknowledging his level and breadth of knowledge, it was also a means of establishing a less authoritative and more democratic and collaborative environment when discussing and in particular in treating the disease. In this instance there was a negotiated acceptance of the characteristics of the disease and its treatment. At the level of physiology, there was also recognition of science's claims of a universal but incomplete ‘truth’ to the extent that much remained unknown or problematic. For example, the biopsy could not absolutely verify the extent of the disease other than to confirm that it existed. However, the hegemony of biomedicine and expert knowledge was confronted at the level of the social and personal.

Power, responsibility and control are important in considering ownership as it applies to illness (Winkelman *et al*. 2005). Healthcare professionals are often inaccurate when assessing patients' preferences for participation in treatment practices. Furthermore, ownership in this context is delegated rather than negotiated. While attempting to consider treatment from the perspective of the patient, the healthcare provider continues to hold power, control and responsibility. This approach is more one-sided in acute or critical care situations and pervades management of life-threatening illness. Nonetheless, this does not suggest that patients do not or are not able to gain some level of ownership over their illness or disease state. For example, experienced patients have been known to take command in interactions with healthcare professionals (Åsbring & Närvänen 2004). While doctors and patients varied in their power of discourse, they could and did hold differing views about appropriate levels of responsibility or control and shared a desire for influence (Åsbring & Närvänen 2004). Therefore, ownership is complex and subject to notions of power and responsibility. Existing with and having identity embedded within the illness or disease provides for moments of psychological vulnerability and losses of personal power. These are often the consequence of ‘not knowing’ and or ‘not understanding’ the disease. Identity is confronted under these circumstances and one looks to overcome and fight. There is a responsibility largely but not limited to one's immediate sense of self and who you are.

Notions of self are constructed and embedded within one's history and associated social and cultural symbols, artefacts and behaviours. Within this symbolic interactionist framework, people interpret situations and take action based upon their view of themselves (Dewar & Lee 2000). The self therefore is not static even though embedded within an historical perspective. Changes in how one perceives one's own identity are a function of the illness or disease states and are encountered continually in everyday life (Kralik *et al*. 2004). Included in these changes are feelings of increased inadequacy and decreased value and influence in the community (Lundwall 2002). Learning to live with a critical illness in particular involves a process of shifts in identity as the individual grapples with the changes in the sense of self that existed prior to the illness. Illness can dominate identity and permeate all aspects of life or effect only part of the self (Kralik *et al*. 2004). These shifts in identity and the struggle for self-preservation involve an ongoing process of negotiation. With prostate cancer, there was the predominately successful attempt to be a part of an everyday ordinary life with an almost unconscious attempt at maintaining an established identity and social roles over the control of symptoms (Townsend *et al*. 2006). It was not particularly given that it was stigmatising. Prostate cancer can largely avoid the stigmatising process as social meaning attached to behaviours or individuals due to such factors as a physical deformity, mental illness or character blemishes (Joachim & Acorn 2000). Nonetheless, the symptoms of prostate cancer can be threatening to identity particularly when involved with incontinence and damaged sexual function.

Diagnosis is also principally disruptive and epiphanic as a moment or point of change. Diagnosis often prompts major life changes. The change can be within or arise from events beyond an individual's control and can be viewed as fate, luck, chance or unexpected opportunity (Thomson *et al*. 2002). Epiphanies can represent opportunities and hazards (King *et al*. 2003) and may be regarded as positive (Whittemore & Roy 2002) or negative. Among Denzin's (1989) four types of epiphanies described as major, cumulative, illuminative and relived, cumulative, epiphanies are linked with chronic illness or disease state as the self is dynamically, continually and progressively reconstituted throughout the prolonged experience of illness (Frank 1995) or disease. Reassessment of identity in this instance lies at the core of illness narrative as epiphanies are pivotal life-changing experiences altering a person's fundamental meaning structures (Wainwright & Turner 2004).

Nonetheless, while seeking precise knowledge of the disease state in terms of its characteristics and its trajectory and path, the initial diagnosis provides limited information. Instead, it unintentionally serves to lessen personal control. In this circumstance, control is with the practitioner although there is meaning in the experience of suffering with the condition (Williams & Koocher 1998). Diagnosis is especially psychologically powerful when it is totally unexpected. Hindsight enabled me to rationalise the increasingly regular need to urinate. However, there was little else that caused concerns of any sort. Consequently, the diagnosis of the probability of having cancer impacted severely. The prominent rational cognitions were confronted by confusion and disbelief. However, while my diagnosis was a surprise, others may legitimise their existing conditions through the diagnosis e.g. multiple sclerosis (Grytten & Maseide 2006). The subsequent attempt to cope may be in the form of self-management to order, control and discipline their lives or alternately to minimise, tolerate, accept or ignore all that cannot be mastered (Kralik *et al*. 2004). Among the forms of self-management or mastery is to ‘intellectualise’ the condition in order to alleviate anxiety (Abram 1980). Social support is also crucial in contending with the disease state and serves as a key mediating factor in the emergence of acceptance and control of illness (Edwards *et al*. 2007).

The extent of trust is also an important element within the cultural and social environment. Trust is based upon the extent to which others (e.g. the healthcare professional or specialist) are able to assist in times of need and distress and is related to hope, perseverance and empathy (Wright 2004). Trusting the expertise, views, opinions and directions of the practitioner facilitates choice and one's awareness of self. However, trust becomes problematic with chronic illness or disease state given the common absence of a clearly defined recovery point (Lee & Poole 2005). Consequently, there is a lack of agency (Diehl *et al*. 2004) in that the person with the illness or disease is unable to assert one's self, to experience competence, achievement, power and mastery in their environment. Under these circumstances, establishing psychological ownership is problematic and in some instances may be considered beyond one's reach. Self-care behaviours are integral in re-establishing ownership through monitoring symptoms, adhering to treatment regimens and engaging in activities that promote health (Regan-Smith *et al*. 2006). In my case, walking and exercising were integral in this process. It was activity in an arena that I could control within the added and presumed assumption of an associated physical and psychological benefit.

## SUMMARY AND CONCLUSIONS

Self-identity is often the subject of greatest change when confronted with an epiphanic experience. It requires one to assess and reassess life and can be confronting as it may also be rewarding. Experiencing a serious illness or being subjected to a life-threatening disease is one of these moments. The effects of the illness may be debilitating and confronting and the illness process often requires one to gain control over its effects by assuming some level of ownership. This does not occur in all instances and a person may choose to have an important other e.g. spouse, medical specialist, to take over this role. However, this role as constructed through social interaction and associated cultural factors may be problematic. This is particularly the case with the medical practitioner and specialist interaction with the person experiencing the disease or illness. Psychological ownership while personal is a socially shared or collective reality that does not retain meaning separated from relationship with others. For example, Beveridge *et al*. (2006) concluded that diabetes appears to be socially owned.

Healthcare professionals need to further embrace the psychological effects of illness and to be aware of and create the psychosocial-cultural environment best suited for enabling the development of a patient's positive self-identity within psychological ownership. Under these circumstances, the positioning of an individual's level of illness or disease ownership is dependent on time, situation and negotiation. It is the product of discourse and associated action between the patient and the practitioner but defined within a broader psychosocial-cultural context. For example, one needs to be aware that a patient will be confronted and will need to interact with a variable group of psychosocio-cultural factors when in an advanced stage of illness or disease than that occupied at the time of diagnosis. Under these circumstances and considering ownership specifically, education or gathering information might gradually alter positioning whereas an epiphany will result in substantial and instant leaps into a new position.

A broader change in the characteristics of the relationship between the practitioner and expert with the person and their disease or illness is necessary to the extent that the process be collaborative and empowering. It is about creating conditions enabling and promoting psychological ownership as an important component in dealing with chronic illness and life-threatening disease. Change is also more likely to be maintained when consistent with what a person believes and desires, when the changes are integrated into the person's sense of self and when the changes are orchestrated in the person's ‘own way’ rather than when motivated by external forces (Bellg 2003). Motivation is a key factor in continuing an engagement with maintaining health-related behaviour. The motivations may be autonomous or controlled with the latter pressured or coerced by an external force (Williams *et al*. 1996). In contrast, healthcare professionals need to help patients internalise and integrate new health behaviours without employing directives (Bellg 2003).
